# High-frequency oscillation and tracheal gas insufflation in patients with severe acute respiratory distress syndrome and traumatic brain injury: an interventional physiological study

**DOI:** 10.1186/cc12815

**Published:** 2013-07-11

**Authors:** Charikleia S Vrettou, Spyros G Zakynthinos, Sotirios Malachias, Spyros D Mentzelopoulos

**Affiliations:** 1 First Department of Intensive Care Medicine, National and Kapodistrian University of Athens Medical School, Evaggelismos General Hospital, Athens, Greece

## Abstract

**Introduction:**

In acute respiratory distress syndrome (ARDS), combined high-frequency oscillation (HFO) and tracheal gas insufflation (TGI) improves gas exchange compared with conventional mechanical ventilation (CMV). We evaluated the effect of HFO-TGI on PaO_2_/fractional inspired O_2 _(FiO_2_) and PaCO_2_, systemic hemodynamics, intracranial pressure (ICP), and cerebral perfusion pressure (CPP) in patients with traumatic brain injury (TBI) and concurrent severe ARDS.

**Methods:**

We studied 13 TBI/ARDS patients requiring anesthesia, hyperosmolar therapy, and ventilation with moderate-to-high CMV-tidal volumes for ICP control. Patients had PaO_2_/FiO_2 _<100 mm Hg at end-expiratory pressure ≥10 cm H_2_O. Patients received consecutive, daily, 12-hour rescue sessions of HFO-TGI interspersed with 12-hour periods of CMV. HFO-TGI was discontinued when the post-HFO-TGI PaO_2_/FiO_2 _exceeded 100 mm Hg for >12 hours. Arterial/central-venous blood gases, hemodynamics, and ICP were recorded before, during (every 4 hours), and after HFO-TGI, and were analyzed by using repeated measures analysis of variance. Respiratory mechanics were assessed before and after HFO-TGI.

**Results:**

Each patient received three to four HFO-TGI sessions (total sessions, *n *= 43). Pre-HFO-TGI PaO_2_/FiO_2 _(mean ± standard deviation (SD): 83.2 ± 15.5 mm Hg) increased on average by approximately 130% to163% during HFO-TGI (*P *< 0.01) and remained improved by approximately 73% after HFO-TGI (*P *< 0.01). Pre-HFO-TGI CMV plateau pressure (30.4 ± 4.5 cm H_2_O) and respiratory compliance (37.8 ± 9.2 ml/cm H_2_O), respectively, improved on average by approximately 7.5% and 20% after HFO-TGI (*P *< 0.01 for both). During HFO-TGI, systemic hemodynamics remained unchanged. Transient improvements were observed after 4 hours of HFO-TGI versus pre-HFO-TGI CMV in PaCO_2 _(37.7 ± 9.9 versus 41.2 ± 10.8 mm Hg; *P *< 0.01), ICP (17.2 ± 5.4 versus 19.7 ± 5.9 mm Hg; *P *< 0.05), and CPP (77.2 ± 14.6 versus 71.9 ± 14.8 mm Hg; *P *< 0.05).

**Conclusions:**

In TBI/ARDS patients, HFO-TGI may improve oxygenation and respiratory mechanics, without adversely affecting PaCO_2_, hemodynamics, or ICP. These findings support the use of HFO-TGI as a rescue ventilatory strategy in patients with severe TBI and imminent oxygenation failure due to severe ARDS.

## Introduction

The management of patients with traumatic brain injury (TBI) becomes challenging when complicated by acute respiratory distress syndrome (ARDS) [1,2]. Hypoxemia, hypercapnia, and hypotension are rather frequent in ARDS, either as original clinical manifestations, or as consequence(s) of the conventional mechanical ventilation (CMV) strategy [3-5]. TBI ventilatory goals include adequate oxygenation as well as CO_2 _elimination for the control of intracranial pressure (ICP) and cerebral perfusion pressure (CPP) [5,6]. However, the use of moderate-to-high tidal volumes and high respiratory rates predisposes TBI patients to ventilator-induced lung injury [4,5].

High-frequency oscillation (HFO) aims at optimizing lung protection [7-10] and recruitment [11]. However, data on the effects of HFO on PaCO_2_, hemodynamics, and ICP in patients with TBI and ARDS are sparse and originate from small, retrospective case series [12-14]. Increases in ICP secondary to transient increases in PaCO_2 _have previously been reported during HFO [12,13]. Hypercapnia occurs commonly during HFO, even at relatively low HFO frequencies of ~5 Hz [15]. Conversely, the addition of tracheal gas insufflation (TGI) to HFO enhances CO_2 _elimination [16,18], and improves oxygenation [16-19]. In the present study, we hypothesized that rescue sessions of HFO-TGI administered to TBI patients with severe ARDS could result in improved gas exchange, higher post-HFO-TGI respiratory compliance, and less-traumatic CMV pressures [19], without adversely affecting ICP and/or CPP.

## Materials and methods

The study was conducted between June 2009 and June 2012 in the mixed medical and surgical 30-bed intensive care unit (ICU) of Evaggelismos Hospital, Athens, Greece. Informed, written next-of-kin consent was obtained for all participants. The study was approved by the Scientific Council and the Ethics Committee of Evaggelismos Hospital.

### Patients

Eligible patients had early (that is, onset within ≤72 hours) ARDS [19,20] with severe oxygenation disturbances (defined as PaO_2_/fractional inspired O_2 _(FiO_2_) ≤ 100 mm Hg at positive end-expiratory pressure (PEEP) ≥10 cm H_2_O), and severe TBI (that is, preintubation Glasgow Coma Score <8 [21]). Target ICP was ≤20 mm Hg; thus, the threshold for increasing therapy-intensity level (TIL) for ICP control was ICP > 20 mm Hg [5,6,22]. TIL comprised a minimum of head elevation (20 degrees to 30 degrees relative to horizontal), higher-dose sedation/neuromuscular blockade, hemodynamic support to maintain a target CPP of ≥60 mm Hg [5,6,22], hyperosmolar therapy, and prevention of hyperthermia ([23]; see also Additional file 1).

We applied previously published exclusion criteria ([19]; Additional file 1), in addition to ICP >30 mm Hg, and brain death or imminent risk of brain herniation. Patient monitoring included continuous display of electrocardiographic lead II and peripheral oxygen saturation, intraarterial blood pressure, cardiac output/index (PICCO-*plus*; Pulsion Medical Systems, Munich, Germany), core patient temperature, and ICP (Codman ICP monitoring system; Codman & Shurtleff, Raynham, MA, USA).

### Study design

**Figure 1:**
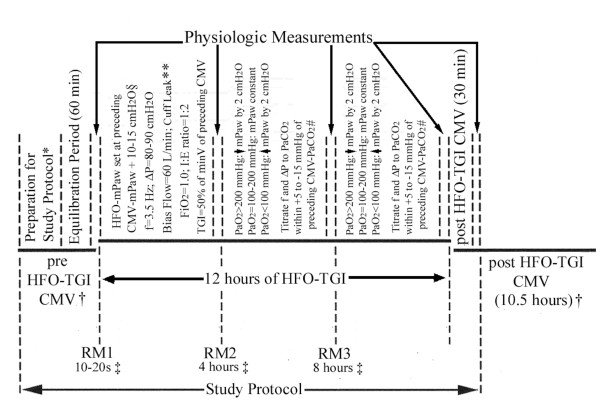
**Schematic representation of the study protocol**. CMV, conventional mechanical ventilation; RM, recruitment maneuver; HFO, high-frequency oscillation; TGI, tracheal gas insufflation; mP_aw_, mean airway pressure; f, oscillation frequency; ΔP, oscillatory pressure amplitude; minV, minute ventilation; FiO_2_, fractional inspired oxygen. *Includes the (1) confirmation of correct positioning and patency of tracheal tubes by chest radiography and 10-second or less fiberoptic endoscopy, respectively [19-21,23]; (2) introduction of a TGI catheter (through a dedicated circuit adapter) and positioning of the TGI catheter tip at 0.5 to 1.0 cm beyond the tracheal tube tip, as previously described [[18-20,22]; Additional file 1]; and (3) minor ventilatory adjustments aimed at further, concurrent optimization of PaCO_2_, intracranial pressure, and plateau pressure (Additional file 1). This patient preparation was carried out once, immediately after study enrolment. †Period duration was as illustrated on study day 1; on a subsequent study day, it constituted a 60-minute pre-HFO-TGI CMV period that followed the 11-hour post-HFO-TGI CMV period of the preceding study day. §Depending on tracheal tube inner diameter (9.0, 8.5, or 8.0 mm) [17], the HFO mP_aw _was set at 10, 12, or 15 cm H_2_O (respectively) above preceding CMV mP_aw _[20]. ‡Performed by pressurizing the HFO breathing circuit at 40 to 45 cm H_2_O for 20 seconds with oscillator piston off. **Causing a 3- to 5-cm H_2_O decrease in mP_aw_, which was reversed by adjusting the mP_aw _valve; the tracheal tube cuff leak was placed immediately after the first RM. #PaCO_2 _of HFO-TGI was to be maintained within 30 to 50 mm Hg.

We conducted a prospective, interventional, noncontrolled study on the physiological effects of intermittent, rescue HFO-TGI in TBI/ARDS patients. In a recent randomized controlled trial of severe ARDS [19], we showed that 6 or more-hour HFO-TGI sessions (average daily HFO-TGI use, 12.4 hours) with recruitment maneuvers (RMs) are associated with significant improvements in oxygenation, plateau pressure, and respiratory compliance during postsession CMV versus presession CMV; HFO-TGI did not significantly affect hemodynamics. Our rescue intervention comprised daily, 12-hour sessions of HFO-TGI and RMs, interspersed with 12-hour periods of CMV (Figure 1). The rescue intervention was discontinued when a PaO_2_/FiO_2 _of >100 mm Hg could be maintained for >12 hours during post-HFO-TGI CMV, with CMV-plateau airway pressure of ≤35 cm H_2_O.

### Study protocol

#### Baseline CMV period

Details are provided in Additional file 1. On enrolment, patients were ventilated with attending physician-prescribed volume assist-control CMV. CMV settings were already titrated to the best possible combinations of PaO_2_/FiO_2 _(target ≥100 mm Hg, with PaO_2 _maintained >90 mm Hg [5,22]), PaCO_2 _(target 35 to 45 mm Hg), plateau pressure (target, ≤35 cm H_2_O), and ICP/CPP. An arterial blood gas analysis was performed, respiratory mechanics were assessed with rapid end-inspiratory/end-expiratory airway occlusion [16-19], and the Murray score [24] was calculated.

Tracheal tube (inner diameter, 8.0 to 9.0 mm) correct positioning and patency were verified, and a circuit adapter/TGI-catheter system was inserted, as previously described [16-19]; Additional file 1. Sixty minutes thereafter, we conducted the study's baseline, physiologic CMV measurements (arterial/central venous blood gas analysis, hemodynamics and ICP, and respiratory mechanics) at FiO_2 _= 1.0 (Figure 1).

#### HFO-TGI and RMs protocol

Patients were connected to the 3100B HFO ventilator (Sensormedics; Yorba Linda, CA, USA), and after a 10- to 20-second period of standard HFO ventilation, a 20-second RM was performed by pressurizing the HFO breathing circuit at 40 to 45 cm H_2_O with the oscillator piston off. RMs were administered only to patients with ICP ≤25 mm Hg and CPP ≥60 mm Hg during pre-HFO-TGI CMV. RM-abort criteria were ICP increase to >25 mm Hg or CPP decrease to <60 mm Hg during an RM; whenever these criteria were met, RMs were suspended until the HFO-TGI session of the next study day.

Initial HFO settings (Figure 1) were aimed at optimizing lung recruitment and PaCO_2 _control. A tracheal tube cuff leak and TGI were used as previously described (Figure 1[16-19]; Additional file 1). For study purposes, we documented physiological measurements (arterial/central venous blood gas analysis, and hemodynamics/ICP) at 4, 8, and 12 hours after HFO initiation. The sequence of RMs, oxygenation-based titrations in mean airway pressure (mP_aw_), and PaCO_2_-based titrations of HFO frequency and oscillatory pressure amplitude (ΔP) is illustrated in Figure 1. If, at 12 hours, the PaO_2_/FiO_2 _was still <100 mm Hg, the daily HFO-TGI session was to be extended for at least 24 hours (that is, until the end of the next day's session [19]).

In the event that ICP would exceed the pre-HFO-TGI value by 5 mm Hg, or reach 30 mm Hg in absolute value for >15 minutes, the HFO-TGI session was to be interrupted, with consequent return to pre-HFO-TGI CMV and cancellation of any further HFO-TGI intervention. During HFO-TGI, any RM-and/or HFO-TGI-associated hypotension (defined as mean arterial pressure <70 mm Hg) lasting for >1 minute was to be treated with norepinephrine and/or a 300 to 500-ml bolus of crystalloid [19].

#### Post HFO-TGI CMV period

If, after 12 hours of HFO-TGI, PaO_2_/FiO_2 _exceeded 100 mm Hg, patients were returned to CMV with the pre-HFO-TGI settings (including the FiO_2 _= 1.0) maintained unchanged for 30 minutes. Subsequently, we performed the post-HFO-TGI physiological measurements. Furthermore, within the next 12 hours, CMV ventilatory settings and TIL for ICP control were retitrated as necessary, in concordance with the previously described targets and limits. Twelve hours after return to CMV, patients were assessed for return to HFO-TGI, according to the previously described, oxygenation/plateau-pressure criterion. The last 60 minutes of this CMV period corresponded to the pre-HFO-TGI CMV period of the subsequent study day (Figure 1). We conducted all daily, pre-HFO-TGI, physiological, CMV measurements with CMV FiO_2 _set at 1.0 for ≥15 minutes.

### Data collection and statistical analysis

On each study day, we obtained physiological measurements over 5-minute periods at the previously mentioned five times (Figure 1). For each 5-minute period, continuously monitored variables were recorded once per minute and then averaged. Standard formula-derived variables included shunt fraction, peripheral O_2 _delivery rate, CPP, respiratory compliance, and oxygenation index (Additional file 1). Daily physiological data sets from each patient were pooled and analyzed.

We conducted a compromise power analysis (G*Power version 3.1; Duesseldorf University, Duesseldorf, Germany), For a small effect size *f *of 0.10, a beta-to-alpha ratio of 4:1, a total of 40 daily data sets (that is, observations), five levels of the within-subjects factor (that is, ventilatory technique), and a nonsphericity correction of 0.3 [17], the analysis yielded an alpha value of 0.044, and a power of 0.83. We estimated that each patient would require three or more HFO-TGI sessions [19], each corresponding to one study-data set [17]. Consequently, a minimum of 13 patients would be required for study completion.

Data were analyzed by using SPSS Statistics version 20 (SPSS Inc., Chicago, IL, USA) and reported as mean ± standard deviation (SD). Distribution normality was tested by using the Kolmogorov-Smirnov test. Physiological variable data obtained at the reported measurement time points were compared with repeated measures analysis of variance for one within-subjects factor. The Bonferroni correction was used for pairwise *post hoc *comparisons. Pre-HFO-TGI and post-HFO-TGI CMV plateau pressure and respiratory-compliance data were compared with a paired *t *test. Significance was set at *P *< 0.05.

## Results

During the study period, we administered rescue HFO-TGI sessions to 13 eligible TBI/ARDS patients. Table 1 displays baseline data of the patients, their Marshall score [25] on hospital admission and their neurologic outcome. On enrolment, six patients had ICP >20 mm Hg and/or CPP <60 mm Hg; average, total TIL score was 17.3 ± 5.1 (range, 11 to 28; Additional file 1, Table S1 [23]). Nine and four patients required a total of three and four daily HFO-TGI sessions (respectively), according to our prespecified oxygenation criteria. No need was seen for extension or interruption of any HFO-TGI session, and none of the HFO-TGI sessions was cancelled. In 13 (30.2%) of 43 HFO-TGI sessions, RMs were cancelled (*n *= 11) or aborted (*n *= 2) (see Additional file 1, Table S2).

**Table 1:** Patient baseline characteristics, ventilatory settings on study enrollment, and outcome

Age (years)	33.1 ± 11.7
Sex (male/female)	9/4
Body mass index (kg/m^2^)	25.0 ± 1.8
PBW (kg)^a^	68.6 ± 8.3
TBI etiology	
Road traffic accident, no/total no (%)	12/13 (92.3)
Fall from height >5 meters, no/total no (%)	1/13 (7.7)
Time from TBI (days)^b^	7.1 ± 1.8
Marshall classification of brain CT findings on hospital admission	
Grade III: Diffuse injury and swelling, no./total no (%)	7/13 (53.9)
Grade VI: Nonevacuated mass lesion >25 ml, no/total no (%)^c, d ^	6/13 (46.2)
Simplified Acute Physiology Score II^e^	48.2 ± 11.9
Thiopental infusion, no/total no (%)^f, g^	4/13 (30.1)
PaO_2_/inspired O_2 _fraction (mm Hg)^f^	85.9 ± 12.2
Fractional inspired O_2_^f^	0.84 ± 0.14
PaCO_2 _(mm Hg)^f^	42.4 ± 15.5
Arterial pH^f^	7.39 ± 0.10
Positive end-expiratory pressure (cm H_2_O)^f^	13.9 ± 2.9
Tidal volume (ml/kg PBW)^f^	8.6 ± 1.8
Respiratory rate (breaths/min)^f^	25.8 ± 6.5
Minute ventilation (L/min)^f^	14.5 ± 2.9
Inspiratory-to-expiratory time ratio^f^	1:2
End-inspiratory plateau airway pressure (cm H_2_O)^f^	33.5 ± 4.7
Mean airway pressure (cm H_2_O)^f^	21.1 ± 2.9
Oxygenation index^f, h^	25.3 ± 3.2
Quasistatic respiratory compliance (ml/cm H_2_O)^f, i^	31.5 ± 6.1
Murray score^f^	3.4 ± 0.4
Time from ARDS diagnosis (hours)^k^	34.9 ± 15.1
Pulmonary ARDS, no/total no (%)^l^	13/13 (100.0)
Outcome according to GOSE	
Upper good recovery (GOSE = 8), no/total no (%)^m^	5/13 (38.5)
Lower good recovery (GOSE = 7), no/total no (%)^m^	2/13 (15.4)
Death (GOSE = 1), no/total no (%)^n^	6/13 (46.2)

Values are mean ± SD unless otherwise specified. TBI, traumatic brain injury; CT, computed tomography; PBW, predicted body weight; ARDS, acute respiratory distress syndrome; GOSE, Glasgow Outcome Scale Extended.

^a^For males, PBW was calculated as 50 + (height (cm) - 152.4) × 0.91; for females, 45.5 + (height(cm) - 152.4) × 0.91.

^b^Refers to the time interval between TBI and study enrollment.

^c^Two patients with epidural hematoma and two patients with subdural hematoma were treated with neurosurgical evacuation within the first 3 hours after hospital admission; on follow-up CT, three patients had diffuse injury III, and one patient (also subjected to decompressive craniectomy) had diffuse injury IV findings.

^d^Two patients with intracerebral hemorrhage received a ventriculostomy; on follow-up CT, one patient had diffuse injury III, and one patient had diffuse injury II findings.

^e^Determined within 12 hours before study enrolment.

^f^Recorded/determined within 10 minutes after study enrolment.

^g^In all four patients, a thiopental infusion of 6 mg/kg/h was started within 24 hours before study enrolment, because their intracranial pressure exceeded 30 mm Hg, despite the preceding combined use of propofol/midazolam anesthesia, hyperosmolar therapy, and increased minute ventilation.

^h^Calculated as mean airway pressure divided by the PaO_2_/inspired O_2 _fraction, and then multiplied by 100.

^i^Calculated as tidal volume divided by the difference between the end-inspiratory and end-expiratory plateau airway pressures.

^k^Refers to the time interval between establishment of ARDS diagnosis and study enrolment.

^l^Eleven patients had severe, bilateral ventilator-associated pneumonia caused by *Klebsiella pneumoniae *(*n *= 5), or *Acinetobacter baumannii *(*n *= 4), or *Pseudomonas aeruginosa *(*n *= 2). Four patients had bilateral pulmonary contusions, and one of them also had a new, unilateral area of consolidation with air-bronchogram, also attributed to ventilator-associated pneumonia with *Acinetobacter baumannii*. One patient also received a massive blood transfusion within the first 48 hours after hospital admission.

^m^Determined at approximately 3 months after hospital discharge; data originate from patient follow-up records of the University-affiliated Department of Neurosurgery of Evaggelismos Hospital.

^n^Corresponds to death in the intensive care unit within 6 to 16 days after study enrolment (see also Table S2 in Additional file 1).

Secondary insults, such as ICP >20 mm Hg, and CPP <60 mm Hg with/without concurrent hypotension, were recorded in 23 (53.5%) of 43 study days corresponding to nine (69.2%) of 13 patients. Insults were effectively treated with further increases in TIL. In all of these cases, at least one insult occurred during CMV. Insults during HFO-TGI were recorded in 19 (44.2%) of 43 study days and in seven (53.8%) of 13 patients (full relevant data reported in Additional file 1, Table S2). This is consistent with the subsequently reported improvements in ICP and CPP control observed during HFO-TGI. In three (7.0%) of 43 study days, concurrent increases in post-HFO-TGI PaCO_2 _(of >5 mm Hg) and ICP (to 23 to 26 mm Hg) were treated mainly by increasing CMV minute ventilation by 1 to 2 L/min (Additional file 1, Supplement to Results and Table S2).

We did not observe any of the potential HFO and/or TGI-associated complications [16-19], apart from transient hypotension within the first 2 minutes of HFO-TGI initiation. This protocol-related complication occurred just after the 20-second first RM in nine (20.9%) of 43 HFO-TGI sessions, corresponding to six (46.2%) of 13 patients. In all cases, the pre-HFO-TGI hemodynamic status was restored within 15 minutes after a temporary increase in vasopressor infusion and a fluid bolus (see Methods and Additional file 1, Supplement to Results and Figure S1).

### Ventilatory parameters and results on physiological variables

We used CMV tidal volume, respiratory rate, minute ventilation, and PEEP of 8.3 ± 1.3 ml/kg predicted body weight, 26.6 ± 5.0 breaths/min, 15.0 ± 2.9 L/min, and 14.6 ± 2.6 cm H_2_O, respectively. Table 2 displays the HFO-TGI settings (along with CMV mP_aw_; see also Figure 1), results on oxygenation index, and CMV respiratory mechanics. HFO-TGI resulted in significant improvements in plateau pressure and respiratory compliance (*P *< 0.01).

**Table 2:** Ventilatory parameters of HFO-TGI sessions, oxygenation index, and respiratory mechanics.

Ventilatory technique	mP_aw _(cm H_2_O)	Frequency (Hz)	ΔP (cm H_2_O)	TGI flow (L/min)	Oxygenation Index	Pplateau (cm H_2_O)	Cst (ml/cm H_2_O)
**Pre HFO-TGI CMV**	20.5 ± 3.1	NA	NA	NA	26.0 ± 8.5	30.4 ± 4.5	37.8 ± 9.2
**HFO-TGI (4 hours)**	31.6 ± 3.9	3.5 ± 0.4	80.9 ± 7.3	3.5 ± 0.4	20.6 ± 10.5*	NA	NA
**HFO-TGI (8 hours)**	30.9 ± 4.3	3.6 ± 0.6	80.4 ± 8.5	3.6 ± 0.8	17.5 ± 7.8*	NA	NA
**HFO-TGI (12 hours)**	30.2 ± 5.0	3.7 ± 0.9	80.1 ± 8.6	3.7 ± 0.9	15.3 ± 5.9*^,§^	NA	NA
**Post HFO-TGI CMV**	19.5 ± 3.0	NA	NA	NA	15.3 ± 5.9*^,§^	28.2 ± 4.6*	45.3 ± 13.1*

Values are mean ± SD. CMV, conventional mechanical ventilation; HFO, high-frequency oscillation; TGI tracheal gas insufflation; pre-HFO-TGI CMV, corresponds to either the baseline CMV period of study day 1 or the 60-minute period that followed the 11-hour period of post-HFO-TGI CMV of the preceding study day (see also Figure 1 and corresponding legend); mP_aw_, mean airway pressure, ΔP, oscillatory pressure amplitude; Pplateau, end-inspiratory plateau airway pressure; C_st_, static respiratory system compliance; NA, not applicable.

*P < 0.01 versus pre-HFO-TGI CMV.

^§ ^*P *< 0.01 versus HFO-TGI at 4 hours.

**Figure 2:**
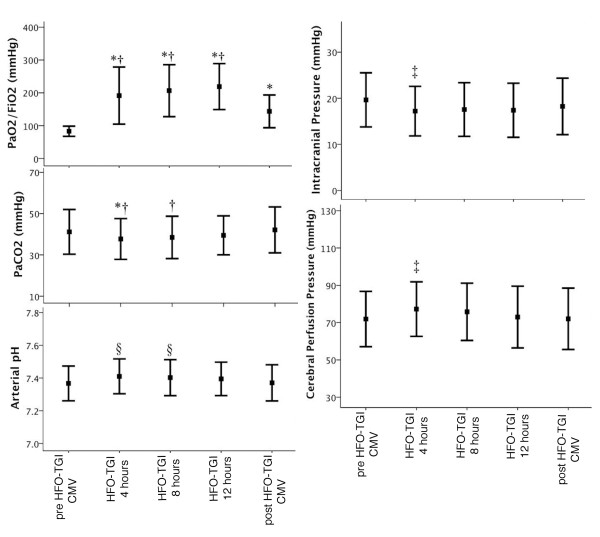
**Results on gas-exchange and cerebral hemodynamics**. CMV, conventional mechanical ventilation; HFO, high-frequency oscillation; TGI, tracheal gas insufflation; pre-HFO-TGI CMV corresponds to either the baseline CMV period of study day 1, or the 60-minute period that followed the 11-hour period of post-HFO-TGI CMV of the preceding study day (see also Figure 1 and corresponding legend). **Left**: results on PaO_2_/fractional inspired oxygen (FiO_2_) (top diagram), PaCO_2 _(middle diagram), and arterial pH (bottom diagram) obtained, during CMV1 (that is, just before HFO-TGI initiation), HFO-TGI at 4, 8, and 12 hours, and CMV2 (that is, at 30 minutes after HFO-TGI discontinuation; see also Figure 1 and corresponding legend). **Right: **results on intracranial pressure (top diagram) and cerebral perfusion pressure (bottom diagram) also obtained at the previously mentioned time points. Squares and error bars represent mean and SD, respectively. *P < 0.01 versus pre-HFO-TGI CMV. †*P *< 0.01 versus post-HFO-TGI CMV. §*P *< 0.05 versus pre-HFO-TGI CMV and post-HFO-TGI CMV. ‡*P *< 0.05 versus pre-HFO-TGI CMV.

Results on PaO_2_/FiO_2_, PaCO_2_, pH, and cerebral hemodynamics are shown in Figure 2. PaO_2_/FiO_2 _was higher during HFO-TGI sessions versus pre-/post-HFO-TGI CMV (*P *< 0.01). Furthermore, PaO_2_/FiO_2 _remained higher during post-HFO-TGI CMV versus pre-HFO-TGI CMV (*P *< 0.01). Accordingly, HFO-TGI was associated with significant improvements in oxygenation index (Table 2), shunt fraction, central-venous O_2 _saturation, and peripheral O_2 _delivery (Table 3). Furthermore, PaCO_2 _and pH were improved after 4 hours of HFO-TGI relative to pre/post HFO-TGI CMV, and after 8 hours of HFO-TGI relative to post-HFO-TGI CMV (Figure 2). ICP and CPP were also improved after 4 hours of HFO-TGI relative to pre/post HFO-TGI CMV (Figure 2). Last, besides the RM-associated hypotension, HFO-TGI did not affect systemic hemodynamics (Table 3).

**Table 3:** Shunt fraction, peripheral perfusion indices, and hemodynamics

Ventilatory strategy	Shunt fraction	ScvO_2 _(%)	Heart rate (beats/min)	MAP (mm Hg)
**Pre HFO-TGI CMV**	0.49 ± 0.09	70.1 ± 6.2	95 ± 24	92 ± 12
**HFO-TGI (4 hours)**	0.31 ± 0.09*	74.0 ± 3.9 *,§	92 ± 23	94 ± 13
**HFO-TGI (8 hours)**	0.29 ± 0.06*	74.6 ± 4.1 *,§	92 ± 23	93 ± 14
**HFO-TGI (12 hours)**	0.29 ± 0.06*	75.0 ± 4.1 *,§	92 ± 22	90 ± 15
**Post HFO-TGI CMV**	0.33 ± 0.14	70.5 ± 6.2	92 ± 22	90 ± 14

**Ventilatory strategy**	**Cardiac Index (L/min/m^2 ^BSA)**	**DO_2 _Index** **(ml/min/m^2 ^BSA)**	**Arterial blood lactate (mM)**	**CVP (mm Hg)**

**Pre HFO-TGI CMV**	4.8 ± 1.3	510 ± 119	1.72 ± 0.70	12 ± 3.4
**HFO-TGI (4 hours)**	4.7 ± 1.1	541 ± 119 §	1.82 ± 0.68	12 ± 3.0
**HFO-TGI (8 hours)**	4.8 ± 1.1	553 ± 114 *,§	1.85 ± 0.68	12 ± 2.9
**HFO-TGI (12 hours)**	4.7 ± 1.2	551 ± 119 *,§	1.82 ± 0.69	12 ± 2.8
**Post HFO-TGI CMV**	4.5 ± 1.1	513 ± 106	1.81 ± 0.74	11.5 ± 3.3

Values are mean ± SD. CMV, conventional mechanical ventilation; HFO, high-frequency oscillation; TGI, tracheal gas insufflation; pre-HFO-TGI CMV, corresponds to either the baseline CMV period of study day 1, or the 60-minute period that followed the 11-hour period of post-HFO-TGI CMV of the preceding study day (see also Figure 1 and corresponding legend); ScvO_2_, central venous O_2 _saturation; MAP, mean arterial pressure; BSA, body surface area; DO_2_, peripheral O_2 _delivery; CVP, central venous pressure.

* P < 0.01 versus pre-HFO-TGI CMV

^§ ^P < 0.05 versus post-HFO-TGI CMV

## Discussion

Our results support the use of HFO-TGI as rescue ventilatory strategy in patients with severe TBI and imminent oxygenation failure due to severe ARDS. In TBI, even a mild arterial hypoxemia (for example, PaO_2 _= 55 to 58 mm Hg) can cause cerebral vasodilation and exacerbation of intracranial hypertension [5,26]. The linear relation between PaCO_2 _and cerebral blood flow and volume [27] mandates control of PaCO_2 _as well.

Current and prior [16-19] results indicate that HFO-TGI substantially improves oxygenation versus CMV. Relative to both CMV and standard HFO, HFO-TGI augments lung base recruitment [16,18]. The high-velocity TGI jet stream likely enhances HFO-dependent gas-transport mechanisms, such as the asymmetry in inspiratory velocity profiles, radial gas mixing, and molecular diffusion [16,17]. TGI also augments dead-space clearance and HFO tidal volume and alveolar ventilation, thereby improving CO_2 _elimination [16,18].

During our current HFO-TGI technique, we used a tracheal tube cuff leak, a high bias flow, and frequency and ΔP settings that correspond to an HFO tidal volume of 180 to 200 ml (Figure 1; Table 2[28]). The latter constitutes a 65% to 67% reduction of the pre-HFO-TGI CMV tidal volume and is consistent with improved lung protection [10]. A better lung protection during post-HFO-TGI CMV relative to pre-HFO-TGI CMV is also suggested by our favorable results on post-HFO-TGI respiratory mechanics (Table 2; [19]).

Assuming a stable chest-wall elastance (E_cw_) during the daily time intervals of the study protocol (Figure 1), the observed increase in respiratory compliance (that is, decrease in respiratory elastance) should reflect a decrease in lung elastance (E_L_) due to HFO-TGI-associated recruitment [16-19]. Also, intrapleural pressure (P_pl_) is given by the equation

$$\text{Ppl}=\text{airway pressure}\times {\text{E}}_{\text{CW}}/({\text{E}}_{\text{L}}+{\text{E}}_{\text{CW}})$$

[29]

This means that for the same airway pressure level and E_cw_, a decrease in E_L _is associated with an increase in P_pl_. Furthermore, in the present study, the average ventilator-displayed HFO mP_aw _during HFO-TGI exceeded the preceding average CMV mP_aw _by about 11 cm H_2_O (Table 2). Consequently, P_pl _was probably increased during HFO-TGI compared with CMV.

An increase in P_pl _could impede systemic and jugular venous return, decrease cardiac output/index and mean arterial pressure, increase ICP, and decrease CPP [30]. In contrast, we observed an initial improvement in cerebral hemodynamics during HFO-TGI (Figure 2). Possible explanatory factors include (a) the mP_aw _decrease along the tracheal tube during HFO-TGI, which results in a mean tracheal pressure that is 5 to 6 cm H_2_O lower than the ventilator-displayed HFO mP_aw _[16,19]; this means that the present study's actual, HFO-TGI-induced increase in average mean tracheal pressure was probably within 5 to 7 cm H_2_O [16]; and (b) an HFO-TGI-induced lung recruitment without concurrent hyperinflation [18]; this is consistent with our favorable results on oxygenation/shunt fraction, and PaCO_2 _(Figure 2 and Table 3).

A prior study of TBI/ARDS [31], showed that ICP and CPP remain stable when an increase in ventilation pressures (through PEEP increase from 0 to 10 cm H_2_O) augments lung recruitment, without affecting PaCO_2_.

Alternative, rescue ventilatory strategies for severe TBI/ARDS patients include prone positioning [5], high-frequency percussive ventilation (HFPV) [5], CMV-TGI [32], pumpless extracorporeal lung assist (pECLA) with a heparin-coated circuit [5,33], and extracorporeal membrane oxygenation (ECMO) [34]. Regarding the use of the first two strategies in TBI/ARDS, only scarce and inconclusive published data exist [5]. CMV-TGI may allow less-traumatic CMV settings while maintaining PaCO_2 _control [32]. CMV-TGI has the limitations of TGI [35], without the option of cuff leak use to lower expiratory airway resistance. pECLA and ECMO may result in better gas exchange and lung protection, with minimal concurrent risk of anticoagulation-induced side effects [5,33,34].

### Methodologic considerations

While designing the study, we anticipated that in severe TBI patients, any new, ARDS-associated hypoxemia and/or hypercapnia could cause reversible ICP perturbations to values >20 mm Hg [5,22]. Furthermore, we considered that an ICP level of 30 mm Hg constitutes an upper limit for its eventual and effective control to ≤20 mm Hg through increases in TIL [36]. Thus, we chose this particular upper ICP limit for both study enrolment and completion of our HFO-TGI intervention. Accordingly, regarding RMs, we chose an upper limit of ICP = 25 mm Hg, because we expected that any potential ICP increase associated with a 20-second RM would most likely be ≤5 mm Hg, thus resulting in a maximal ICP of ≤30 mm Hg during post-RM HFO-TGI [19]. This prediction is consistent with the results of a prior study, which also used ICP >25 mm Hg as the RM-abort criterion [35].

During pressure-controlled CMV, a 60-second RM with an incremental peak pressure of up to 60 cm H_2_O (pressure level sustained for 30 seconds) may decrease mean arterial pressure by about 15% and increase ICP by about ~23%, with concurrent reductions of about 17% in CPP [35]. We applied a continuous positive airway pressure of 40 to 45 cm H_2_O for just 20 seconds. In nine HFO-TGI sessions, the first RMs were associated with average decreases of about 35% and about 44% in mean arterial pressure and CPP (respectively) versus. pre-HFO-TGI CMV; furthermore, within 1 to 2 minutes after RM, the ICP increased by about 19% versus pre-HFO-TGI CMV (see Additional file 1, Figure S1). These protocol-related, secondary insults were promptly reversed by a temporary increase in vasopressor support and volume loading. Insults did not recur after subsequent RMs within the same HFO-TGI session, and occurred independent of session order (Additional file 1, Supplement to Results, and Figure S1). Volume-status optimization may have prevented transient hypotension after the second and third RM of the HFO-TGI sessions [37].

### Study limitations

Limitations of long-term TGI include the impact of the high-velocity jet stream and/or an oscillating TGI catheter on the tracheal wall, causing mucosal necrosis and/or hemorrhage [16-19,38,39], the inspissation of secretions with the potential for partial or complete airway obstruction in case of inadequate humidification of TGI gas [16-19,38,40], and dynamic pulmonary hyperinflation, hemodynamic compromise, and pneumothorax caused by the forward-thrust TGI that can impede expiration [16-19,38]. Other potential complications include venous gas embolism, interference of a TGI catheter passed through the tracheal tube with suctioning [38], TGI catheter obstruction by secretions [19], and absence of commercially available equipment specifically designed for TGI administration [16-19,38]. In our clinical practice, we intermittently superimpose humidified TGI gas to HFO, and most frequently, for ≤12 hours [19]. Furthermore, during HFO-TGI, we use a tracheal tube cuff leak, to increase the effective width of the expiratory pathway, and thus reduce the risk of hyperinflation and promote CO_2 _elimination [8,16-19].

In the present study, the use of brain-tissue O_2 _monitoring could have clarified the relation between the HFO-TGI-induced improvement in arterial oxygenation and the oxygenation of the brain tissue. It would have also have been of great interest to include transcranial Doppler ultrasonography measurements as part of the trial, to investigate the effect of HFO-TGI on cerebral blood flow. Finally, the study was noncontrolled and nonrandomized. However, it provides the first supporting data on the feasibility, efficacy, and safety of HFO-TGI in severe TBI/ARDS.

## Conclusions

HFO-TGI improves oxygenation and lung mechanics and does not adversely affect hemodynamics, CO_2 _elimination, ICP, and CPP when used to ventilate TBI patients with severe ARDS. RMs can cause hemodynamic complications and may have to be cancelled or aborted.

## Key messages

• The use of HFO in patients with TBI is limited because of hypercapnia that occurs commonly during HFO, even at relatively low HFO frequencies of about5 Hz. Hypercapnia can have deleterious effects on ICP and CPP.

• The addition of TGI to HFO improves oxygenation and enhances CO_2 _elimination, thereby providing a theoretically suitable lung-protective strategy for patients with ARDS/TBI.

• In this work, we showed that rescue sessions of HFO-TGI administered to TBI patients with severe ARDS result in improved gas exchange, higher post-HFO-TGI respiratory compliance, and less-traumatic CMV pressures, without adversely affecting ICP and/or CPP.

• Our findings support the design of randomized controlled trials to evaluate the use of HFO-TGI in patients with ARDS and TBI.

## Abbreviations

ARDS: acute respiratory distress syndrome; CMV: conventional mechanical ventilation; CPP: cerebral perfusion pressure; ECMO: extracorporeal membrane oxygenation; E_cw_: chest wall elastance; E_L_: lung elastance; FiO_2_: fractional inspired O_2_; HFO: high-frequency oscillation; HFPV: high-frequency percussive ventilation; ICP: intracranial pressure; mP_aw_: mean airway pressure; pECLA: pumpless extracorporeal lung assist; PEEP: positive end-expiratory pressure; P_pl_: intrapleural pressure; RM: recruitment maneuver; TBI: traumatic brain injury; TGI: tracheal gas insufflation; TIL: therapy intensity level; ΔP: oscillatory pressure amplitude.

## Competing interests

The authors declare that they have no competing interests.

## Authors' contributions

CSV, SGZ, and SDM contributed to the conception and design of the study. SDM and SMa collected the data. CSV and SDM analyzed and interpreted the data. All authors contributed to the discussion of the results. CSV and SMa drafted the manuscript, and SGZ and SDM critically revised it. All authors read and approved the final manuscript for publication.

## Supplementary Material

Additional file 1 **Electronic Supplementary Material to High-Frequency Oscillation and tracheal gas insufflation in patients with severe acute respiratory distress syndrome and traumatic brain injury: An interventional physiological study**. Details of methods and data not shown in the main manuscript. Click here for file
