# Supplementary material for: High-frequency oscillation and tracheal gas insufflation in patients with severe acute respiratory distress syndrome and traumatic brain injury: an interventional physiological study
Source: Crit Care. 2013 Jul 11;17(4):R136. doi: 10.1186/cc12815 (PMC4057500; doi:10.1186/cc12815)
Supplement: Additional file 1 — Electronic Supplementary Material to High-Frequency Oscillation and tracheal gas insufflation in patients with severe acute respiratory distress syndrome and traumatic brain injury: An interventional physiological study. Details of methods and data not shown in the main manuscript. [file cc12815-S1.DOC]

**Electronic Supplementary Material to:**

**High frequency oscillation and tracheal gas insufflation in patients with severe acute respiratory distress syndrome and traumatic brain injury: An interventional physiological study**

**Authors**

1. Charikleia Vrettou, MD, Consultant in Intensive Care Medicine^1^
2. Spyros G. Zakynthinos, MD, PhD, Professor in Intensive Care Medicine^1^
3. Sotirios Malachias, MD, Consultant in Intensive Care Medicine^1^
4. Spyros D. Mentzelopoulos, MD, PhD, Assistant Professor in Intensive Care Medicine^1^*

1) First Department of Intensive Care Medicine, National and Kapodistrian University of Athens Medical School, Evaggelismos General Hospital, Athens, Greece.

*Corresponding author: Spyros D. Mentzelopoulos, MD, PhD, Mailing Address: Department of Intensive Care Medicine, University of Athens Medical School, Evaggelismos General Hospital, 45-47 Ipsilandou Street, GR-10676, Athens, Greece. E-mail: [sdmentzelopoulos@yahoo.com](mailto:sdmentzelopoulos@yahoo.com); [sdm@hol.gr](mailto:sdm@hol.gr). Phone: +306975304909.

**Supplement to Methods**

**Exclusion Criteria**

In addition to intracranial pressure (ICP)>30 mmHg, and brain death or imminent risk of brain herniation, exclusion criteria were age<18 or >75 years, active air leak or recent severe air leak (i.e. >1 chest tube per hemithorax with persistent gas leak for >72 hours), severe hemodynamic instability (i.e. systolic arterial pressure<90 mmHg, despite volume loading and norepinephrine infusion at ≥0.5 μg/kg/min), significant heart disease or obstructive lung disease, pregnancy, and morbid obesity (i.e. body mass index>40 kg/m^2^) [1].

*Patient preparation during the baseline conventional mechanical ventilation (CMV) period (see also Methods and Figure 1 of the main manuscript)*

Following study enrolment, orotracheal tubes (inner diameter=8.0-9.0 mm) were cut-down to 26 cm, correct positioning of tracheal tube tip (~4 cm above the carina) was verified by chest radiography, and tracheal tube patency was confirmed by a ≤10-sec-lasting endoscopy using an intubating fiberscope passed through the catheter mount of the breathing circuit [1-4]. A 4.8-cm long, circuit adapter with angled side arms (Smiths Medical International, Watford, UK) was introduced in-between tracheal tube connector and Y-piece of the ventilator breathing circuit. A rigid-wall catheter (Vygon, Ecouen, France; inner diameter=1.0 mm, outer diameter=2.0 mm) was passed through the side arm of the proximal adapter. The catheter was used for the administration of tracheal gas insufflation (TGI) during the high frequency oscillation (HFO)-TGI sessions. TGI catheter length was tailored to the placement of its tip at 0.5-1.0 cm beyond the tip of the tracheal tube [1-4]. Subsequently, we performed minor adjustments in tidal volume and respiratory rate aimed at further, concurrent optimization of PaCO_2_, ICP, and plateau pressure (data not shown). Sixty min thereafter, we conducted the study’s baseline, physiologic CMV measurements at FiO_2_=1.0. In patients ventilated at FiO_2_<1.0 on enrollment we set CMV FiO_2_ at 1.0 at 15 min before the baseline measurements.

*Addition of Tracheal tube cuff leak and TGI to HFO*

We connected the patients to the HFO ventilator, and after 10-20 s of standard HFO ventilation, we administered a recruitment maneuver (see also Methods and Figure 1 of the main manuscript). We then resumed HFO and placed a 3-5 cmH_2_O tracheal tube cuff leak. We returned mean airway pressure (mPaw) to its pre-leak level by adjusting the mPaw valve. Subsequently, we connected the TGI catheter to a variable-orifice O_2_ flowmeter providing humidified O_2_ at room temperature, and started TGI at a flow equal to 50% of the preceding CMV minute ventilation [1-4; see also Figure 1 of the main manuscript]. TGI initiation caused a 1-2-cmH_2_O rise in mPaw, which we reversed by adjusting the mPaw valve [1-4].

**Supplement to Results**

During the study period, 87 patients were admitted for traumatic brain injury (TBI ) with/without other concurrent injuries to our hospital’s intensive care unit. Twenty six TBI patients developed ARDS [5], and 17 of these patients fulfilled our combination eligibility criterion of severe TBI and severe ARDS (see Methods of the main manuscript). We excluded 2 patients due to severe pulmonary air leak, and another 2 patients due to severe hemodynamic instability. Thus, we ultimately applied our rescue HFO-TGI protocol to 13 patients.

Table S1 displays full data on the baseline therapy intensity level (TIL) for ICP control [6]. Table S2 displays detailed, individual patient data on secondary insults {i.e. ICP>20 mmHg, cerebral perfusion pressure (CPP)<60 mmHg, and mean arterial pressure<70 mmHg} and corresponding adjustments in TIL, and neurological outcome.

All study participants required vasopressor support throughout the study period. The pre HFO-TGI infusion rates of norepinephrine were respectively 0.20±0.04, 0.21±0.07, 0.20±0.07, and 0.25±0.10 μg/kg/min on days 1, 2, 3, and 4. For days 1-3, data originate from all 13 study participants, whereas for day 4, data originate from the 4 patients, who required 4 sessions of HFO-TGI (see also Results of the main manuscript). On each study day, these pre HFO-TGI norepinephrine infusion rates were the same at the time points of the HFO-TGI and post HFO-TGI measurements. The same was true for the additional vasopressin/dobutamine and vasopressin support required in the second and seventh enrollee, respectively (Table S2).

Table S2 also shows that the secondary insults and corresponding adjustments in TIL occurred mainly during CMV. In 3/43 study days (7.0%) corresponding to 2 patients, we noted post HFO-TGI ICP perturbations (i.e. rises to 23-26 mmHg) attributable to PaCO_2_ rises of >5 mmHg relative to the end of the HFO-TGI session; these particular insults were treated by increases in TIL that also included increases in CMV minute ventilation by 1-2 L/min (Table S2).

During the time intervals between the first and last set of the daily physiologic measurements of each study day, there were no remarkable changes in physiologic stress level and core body temperature (Table S1 and Table S2), feeding, and medical treatment (detailed data not shown). However, the stability of major determinants of energy expenditure or metabolic rate implies a stable CO_2_ production [3]. Furthermore, cardiac index (see also Table 3 of the main manuscript) and estimated central-venous CO_2_ concentration {3; data not shown] were also stable, indicating a stable CO_2_ delivery rate to the lungs [3]. Consequently, differences in PaCO_2_ between time points of physiologic measurements (see also Figure 2 of the main manuscript) primarily reflected changes in CO_2_ elimination efficiency [3].

Recruitment maneuvers (RMs) were not performed in 11 of the study’s 43 HFO-TGI sessions due to CPP<60 mmHg (n=4), or ICP>25 mmHg (n=1), or CPP<60 mmHg and ICP>25 mmHg (n=6) during pre HFO-TGI CMV (Table S2). RM cancellation criteria were fulfilled once or twice by 7 patients. Furthermore, in 1 patient, the first RMs of 2 consecutive HFO-TGI sessions were aborted, because ICP exceeded 25 mmHg (by 1-2 mmHg) during their administration (Table S2); in both sessions, ICP dropped to <25 mmHg within 30 s following resumption of HFO-TGI ventilation. Consequently, RMs were cancelled or aborted in 13/43 HFO-TGI sessions (30.2%), and 91 RMs were actually administered according to study protocol (see also Figure 1 of the main manuscript).

Figure S1 displays individual patient data on RM-associated hypotension. This, protocol-related complication occurred just after the first RM in 9/43 HFO-TGI sessions. In all cases, the pre HFO-TGI mean arterial pressure level was promptly (i.e. within 8.7±3.6 min of resumption of HFO-TGI ventilation) restored with increases of 20-30% in norepinephrine infusion rates. The latter were also restored to their pre-HFO-TGI levels within the first 60 min of the HFO-TGI sessions, after the administration of 300-500 mL of crystalloid. These transient hypotensive episodes did not seem to have affected patient outcomes. According to the Glasgow Outcome Scale Extended, patients nos 1, 3, 4, 12, and 13 (Figure S1) had an upper good recovery, and patient no. 10 (Figure S1) had a lower good recovery.

**Appendix**

**Derived physiological variables**

These variables were derived according to the following formulas:

1. Oxygenation Index = 100 x mPaw x FiO_2_/PaO_2_

2. Quasistatic Respiratory System Compliance = Vt/(Pplateau– PEEPtot)

3. CaO_2_ = Hgb x 1.36 x SaO_2_ / 10 + 0.003 x PaO_2_

4. CcvO_2_ = Hgb x 1.36 x ScvO_2_ / 10 + 0.003 x PcvO_2_

5. PAO_2_ = PiO_2_-PACO_2_ x [FiO_2_-(1-FiO_2_) / R]; PiO_2_ = FiO_2_ x (PB-47);

PACO_2_ ~ PaCO_2_;

R = (FEY of Carbohydrate Intake) x 1.0 + (FEY of Protein Intake) x 0.8 +

(FEY of Lipid Intake) x 0.7.

6. CcO_2_ = Hgb x 1.36 / 10 + 0.003 x PAO_2_

7. Shunt Fraction = (CcO_2_-CaO_2_) / (CcO_2_-CcvO_2_)

8. O_2_ Delivery Index = Cardiac Index x CaO_2_

9. Cerebral Perfusion Pressure = Mean Arterial Pressure – Intracranial Pressure

Where mPaw = mean airway pressure (cmH_2_O); Vt = tidal volume (mL); Pplateau = end-inspiratory plateau airway pressure (cmH_2_O); PEEPtot = end-expiratory plateau airway pressure (cmH_2_O); FiO2 = inspired O_2_ fraction; Pa, Pcv, PA, and Pi = arterial, central-venous, alveolar, and inspired gas partial pressure (mmHg), respectively; CaO_2_, CcvO_2_, and CcO_2_ = O_2_ content in arterial, central-venous, and pulmonary end-capillary blood (mL), respectively; Hgb = hemoglobin concentration (g/L); 1.36 = O_2_ combining power of 1 g of hemoglobin (mL); SaO_2_ and ScvO_2_ = arterial and central-venous O_2_ saturation as determined by the blood-gas analyzer, respectively; 0.003 = O_2_ solubility coefficient at 37 °C (mL x mmHg/dL); R = respiratory quotient; P_B_, barometric pressure (mmHg); 47 = H_2_O saturated vapor pressure at 37 °C (mmHg); FEY = fractional energy yield relative to total of prescribed nutritional support; CI = cardiac index (L/min/m^2^ body surface area). For the computation of shunt fraction, we used blood gas values obtained from the central-venous blood [1-4].

**References**

1. Mentzelopoulos SD, Malachias S, Zintzaras E, Kokkoris S, Zakynthinos E, Makris D, Magira E, Markaki V, Roussos C, Zakynthinos SG: **Intermittent recruitment with high-frequency oscillation/tracheal gas insufflation in acute respiratory distress syndrome**. *Eur Respir J* 2012, **39:** 635–647.
2. Mentzelopoulos SD, Roussos C, Koutsoukou A, Sourlas S, Malachias S, Lachana A, Zakynthinos SG: **Acute effects of combined high- frequency oscillation and tracheal gas insufflation in severe acute respiratory distress syndrome.** *Crit Care Med* 2007, **35:**1500–1508.
3. [Mentzelopoulos SD](http://www.ncbi.nlm.nih.gov/pubmed?term=Mentzelopoulos SD%5BAuthor%5D&cauthor=true&cauthor_uid=20232047), [Malachias S](http://www.ncbi.nlm.nih.gov/pubmed?term=Malachias S%5BAuthor%5D&cauthor=true&cauthor_uid=20232047), [Kokkoris S](http://www.ncbi.nlm.nih.gov/pubmed?term=Kokkoris S%5BAuthor%5D&cauthor=true&cauthor_uid=20232047), [Roussos C](http://www.ncbi.nlm.nih.gov/pubmed?term=Roussos C%5BAuthor%5D&cauthor=true&cauthor_uid=20232047), [Zakynthinos SG](http://www.ncbi.nlm.nih.gov/pubmed?term=Zakynthinos SG%5BAuthor%5D&cauthor=true&cauthor_uid=20232047): **Comparison of high-frequency oscillation and tracheal gas insufflation versus standard high-frequency oscillation at two levels of tracheal pressure.** *[Intensive Care Med](http://www.ncbi.nlm.nih.gov/pubmed/20232047#%23)* 2010, **36:**810-816.
4. [Mentzelopoulos SD](http://www.ncbi.nlm.nih.gov/pubmed?term=Mentzelopoulos SD%5BAuthor%5D&cauthor=true&cauthor_uid=21369813), [Theodoridou M](http://www.ncbi.nlm.nih.gov/pubmed?term=Theodoridou M%5BAuthor%5D&cauthor=true&cauthor_uid=21369813), [Malachias S](http://www.ncbi.nlm.nih.gov/pubmed?term=Malachias S%5BAuthor%5D&cauthor=true&cauthor_uid=21369813), [Sourlas S](http://www.ncbi.nlm.nih.gov/pubmed?term=Sourlas S%5BAuthor%5D&cauthor=true&cauthor_uid=21369813), [Exarchos DN](http://www.ncbi.nlm.nih.gov/pubmed?term=Exarchos DN%5BAuthor%5D&cauthor=true&cauthor_uid=21369813), [Chondros D](http://www.ncbi.nlm.nih.gov/pubmed?term=Chondros D%5BAuthor%5D&cauthor=true&cauthor_uid=21369813), [Roussos C](http://www.ncbi.nlm.nih.gov/pubmed?term=Roussos C%5BAuthor%5D&cauthor=true&cauthor_uid=21369813), [Zakynthinos SG](http://www.ncbi.nlm.nih.gov/pubmed?term=Zakynthinos SG%5BAuthor%5D&cauthor=true&cauthor_uid=21369813): **Scanographic comparison of high frequency oscillation with versus without tracheal gas insufflation in acute respiratory distress syndrome.** *Intensive Care Med* 2011, **37:**990-999.
5. The ARDS Definition Task Force: **Acute Respiratory Distress Syndrome. The Berlin Definition.** *JAMA* 2012, **307:**2526-2533.
6. **Therapy Intensity Level** [http://[www.tbi-impact.org/cde/mod_templates/T_TIL.9.1.pdf](http://www.tbi-impact.org/cde/mod_templates/T_TIL.9.1.pdf) ].
7. [Johnston AJ](http://www.ncbi.nlm.nih.gov/pubmed?term=Johnston AJ%5BAuthor%5D&cauthor=true&cauthor_uid=14633744), [Steiner LA](http://www.ncbi.nlm.nih.gov/pubmed?term=Steiner LA%5BAuthor%5D&cauthor=true&cauthor_uid=14633744), [Chatfield DA](http://www.ncbi.nlm.nih.gov/pubmed?term=Chatfield DA%5BAuthor%5D&cauthor=true&cauthor_uid=14633744), [Coleman MR](http://www.ncbi.nlm.nih.gov/pubmed?term=Coleman MR%5BAuthor%5D&cauthor=true&cauthor_uid=14633744), [Coles JP](http://www.ncbi.nlm.nih.gov/pubmed?term=Coles JP%5BAuthor%5D&cauthor=true&cauthor_uid=14633744), [Al-Rawi PG](http://www.ncbi.nlm.nih.gov/pubmed?term=Al-Rawi PG%5BAuthor%5D&cauthor=true&cauthor_uid=14633744), [Menon DK](http://www.ncbi.nlm.nih.gov/pubmed?term=Menon DK%5BAuthor%5D&cauthor=true&cauthor_uid=14633744), [Gupta AK](http://www.ncbi.nlm.nih.gov/pubmed?term=Gupta AK%5BAuthor%5D&cauthor=true&cauthor_uid=14633744): [**Effects of propofol on cerebral oxygenation and metabolism after head injury.**](http://www.ncbi.nlm.nih.gov/pubmed/14633744) *Br J Anaesth* 2003, **91:**781-786.

**Table S1.** Scoring of the therapy intensity level (TIL) used for the achievement and/or maintenance of an intracranial pressure (ICP) of ≤20 mmHg on study enrolment.

| **Patient number** | | | | | | | | | | | | | |
| --- | --- | --- | --- | --- | --- | --- | --- | --- | --- | --- | --- | --- | --- |
| **TIL subcomponent** | 1 | 2^a^ | 3 | 4 | 5 ^a^ | 6 | 7 ^a^ | 8 ^a^ | 9 | 10 ^a^ | 11 | 12 | 13 ^a^ |
| Head elevation | 1 | 1 | 1 | 1 | 1 | 1 | 1 | 1 | 1 | 1 | 1 | 1 | 1 |
| Sedation (higher dose) | 2 | 0 | 2 | 2 | 0 | 0 | 0 | 2 | 2 | 2 | 2 | 2 | 0 |
| Metabolic suppression^b^ | 0 | 5 | 0 | 0 | 5 | 5 | 5 | 0 | 0 | 0 | 0 | 0 | 5 |
| NM blockade | 3 | 3 | 3 | 3 | 3 | 3 | 3 | 3 | 3 | 3 | 3 | 3 | 3 |
| CSF drainage ≤5 mL/h | 0 | 0 | 0 | 0 | 0 | 0 | 2 | 0 | 2 | 0 | 0 | 0 | 0 |
| CSF drainage >5 mL/h | 0 | 0 | 0 | 0 | 0 | 0 | 0 | 0 | 0 | 0 | 0 | 0 | 0 |
| Fluid loading^c^ | 1 | 1 | 1 | 1 | 1 | 1 | 1 | 1 | 1 | 1 | 1 | 1 | 1 |
| Norepinephrine infusion^c^ | 1 | 1 | 1 | 1 | 1 | 1 | 1 | 1 | 1 | 1 | 1 | 1 | 1 |
| PaCO_2_ of 35-40 mmHg | 1 | 0 | 0 | 0 | 0 | 1 | 0 | 0 | 0 | 0 | 1 | 0 | 0 |
| PaCO_2_ of 30-35 mmHg | 0 | 0 | 2 | 2 | 0 | 0 | 0 | 0 | 2 | 0 | 0 | 2 | 0 |
| PaCO_2_ of <30 mmHg | 0 | 0 | 0 | 0 | 4 | 0 | 0 | 0 | 0 | 0 | 0 | 0 | 0 |
| Mannitol <2 g/kg/24 h^d^ | 2 | 0 | 2 | 2 | 2 | 2 | 2 | 2 | 2 | 0 | 2 | 0 | 2 |
| Mannitol ≥2 g/kg/24 h^d^ | 0 | 3 | 0 | 0 | 0 | 0 | 0 | 0 | 0 | 3 | 0 | 0 | 0 |
| HT 3% saline <0.3 g/kg/24 h^d^ | 2 | 0 | 0 | 2 | 2 | 2 | 2 | 0 | 2 | 0 | 0 | 2 | 0 |
| HT 3% saline ≥0.3 g/kg/24 h^d^ | 0 | 3 | 0 | 0 | 0 | 0 | 0 | 0 | 0 | 3 | 0 | 0 | 0 |
| Treatment of fever^e^ | 1 | 1 | 1 | 1 | 0 | 0 | 0 | 1 | 1 | 0 | 1 | 1 | 1 |
| Mild hypothermia up to 35 °C | 0 | 0 | 0 | 0 | 2 | 0 | 0 | 0 | 0 | 0 | 0 | 0 | 0 |
| Hypothermia (33-34 °C) | 0 | 0 | 0 | 0 | 0 | 5 | 5 | 0 | 0 | 5 | 0 | 0 | 0 |
| Intracranial operation^f^ | 0 | 4 | 0 | 0 | 0 | 0 | 0 | 0 | 0 | 0 | 0 | 0 | 0 |
| Decompressive Craniectomy^g^ | 0 | 6 | 0 | 0 | 0 | 0 | 0 | 0 | 0 | 0 | 0 | 0 | 0 |
| **Total TIL Score** | **14** | **28** | **13** | **15** | **21** | **21** | **22** | **11** | **17** | **22** | **12** | **13** | **16** |

The total TIL score is the vertical sum of the subscores corresponding to the TIL subcomponents. NM, neuromuscular; CSF, cerebrospinal fluid; HT, hypertonic.

^a^, TIL increased within 3 hours pre-enrolment due to ICP>20 mmHg.

^b^, Either a thiopental infusion of up to 6.0 mg/kg/h (patients nos. 2, 5, 7, and 10) or a propofol infusion of 4.3 mg/kg/h (patient no. 6 with concurrent midazolam infusion of 0.2 mg/kg/h) were used to achieve an electroencephalographic burst-suppression {defined as bursts of high amplitude theta/delta activity with intervening periods of electrical quiescence [7]}, in conjunction with an ICP of ≤20 mmHg.

^c^, Used for the maintenance of a cerebral perfusion pressure of ≥60 mmHg; hemodynamic support was anyway required due to ventilator-associated pneumonia-induced septic shock in 12 of the 13 patients or recovery from hemorrhagic shock following surgical control of intra-abdominal bleeding in patient no. 3.

^d^, Hyperosmolar therapy was to be continued as prescribed, provided that serum osmolarity did not exceed 320 mOsm/kg H_2_O.

^e^, Defined as core body temperature >38º C; fever was treated with conventional antipyretics or cooling measures; a spontaneous core body temperature of <34.5 º C also caused an increase of TIL by 1 point.

^f^, Not scheduled on hospital admission and indicated for the management of a progressive mass lesion; in the particular case of patient no. 2, there was an expanding subdural hematoma (from <0.5 cm thickness to ~1 cm thickness) confirmed on the first follow-up computerized tomography (CT) of the brain; this follow-up CT was performed at 24 h following hospital admission.

^g^, Performed ~48 hours before study enrolment

**Table S2. Data on secondary insults and outcome**.

| **Patient No. /Day of**  **Secondary insult** | **Type, protocol sub-period, and duration of insult** | **Worst ICP and/or CPP values recorded during the designated study day sub-period(s)** | **Treatment of insult** | **GOSE^a^** |
| --- | --- | --- | --- | --- |
| **1 / study day 3** | ICP>20 mmHg & CPP<60 mmHg for <15 min^b^ during CMV2 | ICP=21 mmHg & CPP=57 mmHg (during CMV2) | ↑ Propofol infusion rate from 2.6 to 3.3 mg/kg/h during CMV2 | 8: Upper good recovery |
| **2 / study day1**  **2 / study day 2**  **2 / study day 3** | ICP>20 mmHg & CPP<60 mmHg during CMV1, the last 4 hours of the HFO-TGI session, and CMV2 – Vasopressor-refractory septic shock^d^ ; **RMs of HFO-TGI session cancelled**^c^  ICP>20 mmHg & CPP<60 mmHg during CMV1 and HFO-TGI; CPP<60 mmHg & MAP most frequently within 60-65 mmHg throughout day 2 – Vasopressor-refractory septic shock^d^; **RMs of HFO-TGI session cancelled** ^c^  ICP>20 mmHg for <15 min during HFO-TGI; ICP>20 mmHg & CPP<60 mmHg for <15 min during CMV2 | ICP=26 mmHg & CPP=48 mmHg (during CMV2)  ICP=23 mmHg^i^ & CPP=25 mmHg (during CMV2), with MAP=48 mmHg  ICP=23 mmHg^i^ & CPP=56 mmHg (during CMV2) | Hypothermia (33-34 °C) instituted during CMV1; Crystalloid bolus of 500 mL during CMV1 and CMV2; Addition of Dobutamine at 5 μg/kg/min and of Vasopressin at 0.02 IU/min^d^ during CMV2  ↑ Norepinephrine infusion rate from 0.27 to 0.3 μg/kg/min during CMV1; ↓ of HFO frequency to 3.0-3.2 Hz & ↑ of CMV minute ventilation by 2 L/min to improve PaCO_2_ control during CMV2; ↑ Vasopressin infusion rate from 0.02 to 0.04 IU/min^d^ & Crystalloid bolus of 1000 mL of during CMV2  ↓ PaCO_2_ within 30-35 mmHg during HFO-TGI; ↑ of CMV minute ventilation by 1 L/min to improve PaCO_2_ control during CMV2 | 1: Death of septic shock and MOF at 11 days post-enrolment |
| **3 / study day 1**  **3 / study day 2**  **3 / study day 3**  **3 / study day 4** | CPP<60 mmHg for <15min during HFO-TGI; ICP>20 mmHg during CMV2 for <60min^e^  ICP>20 mmHg throughout day 2, apart from first 4 hours of HFO-TGI; **First RM of HFO-TGI session aborted due to ICP >25 mmHg**  ICP>20 mmHg during CMV1 and first 4 hours of HFO-TGI; **First RM of HFO-TGI session aborted due to ICP >25 mmHg**  ICP>20 mmHg for <60 min during both HFO-TGI and CMV2 | CPP=58 mmHg during CMV2 (while ICP≤20); ICP=23 mmHg during CMV2 (while CPP≥60)  ICP=24 mmHg during CMV1 (while CPP≥60)  ICP=24 mmHg during CMV1 (while CPP≥60)  ICP=24 mmHg during CMV2 (while CPP≥60) | Addition of HT 3% saline at 0.1 g/kg/24 h during CMV2  ↑ Thiopental infusion rate from 6.0 to 7.0 mg/kg/h during CMV1  ↑ in HT 3% saline to 0.32 g/kg/24 h during CMV1  Thiopental bolus of 250 mg given once during HFO-TGI and once during CMV2 | 1: Death of septic shock and MOF at 6 days post-enrolment |

| **Patient No. /Day of**  **Secondary insult** | **Type, protocol sub-period, and duration of insult** | **Worst ICP and/or CPP values recorded during the designated study day sub-period(s)** | **Treatment of insult** | **GOSE^a^** |
| --- | --- | --- | --- | --- |
| **5 / study day 1**  **5 / study day 3**  **5 / study day 4** | CPP<60 mmHg during CMV1; ICP>20 mmHg throughout day 1; **RMs of HFO-TGI session cancelled** ^f^  ICP>20 mmHg throughout day 3  ICP>20 mmHg throughout day 4; CPP<60 mmHg for <15 min during both HFO-TGI and for <60 min CMV2; **RMs of HFO-TGI session cancelled** ^g^ | CPP=40 mmHg during CMV1 (while ICP≤20); ICP=27 mmHg during CMV2 (while CPP≥60)  ICP=23 mmHg during CMV2 (while CPP≥60)  ICP=30 mmHg during CMV1 (while CPP≥60); CPP=50 mmHg & ICP=27 mmHg during CMV2 | ↑ Norepinephrine infusion rate from 0.22 to 0.26 μg/kg/min &  ↑ HT saline to >0.3 g/kg/24 h during CMV1; ↑ Mannitol to >2 g/kg/24 h during CMV2  ↑ Thiopental infusion rate from 6.0 to 6.5 mg/kg/h during CMV1  ↑ Thiopental infusion rate from 6.5 to 7.5 mg/kg/h during CMV1; ↑ Norepinephrine infusion rate from 0.30 to 0.36 μg/kg/min during CMV2 | 1: Death of septic shock and MOF at 8 days post-enrolment |
| **6 / study day 1**  **6 / study day 2**  **6 / study day 3** | ICP>20 mmHg throughout day 1, apart from first 4 hours of HFO-TGI  ICP >20 mmHg during HFO-TGI and CMV2  ICP>20 mmHg during CMV1 and first 8 hours of HFO-TGI; CPP<60 mmHg for <60min during CMV1 and for <15 min during HFO-TGI ; **RMs of HFO-TGI session cancelled**^f^ | ICP=40 mmHg^k^ during CMV2 (while CPP≥60)  ICP=33 mmHg^k^ during CMV2 (while CPP≥60)  ICP=23 mmHg & CPP=53 mmHg (during CMV1); ICP=22 mmHg & CPP=49 mmHg (during HFO-TGI); ICPmax=25 mmHg during HFO-TGI (while CPP≥60) | ↑ Norepinephrine infusion rate from 0.18 to 0.24 μg/kg/min & Thiopental bolus of 250 mg & ↑ Propofol infusion rate to 5.0 mg/kg/h during CMV2  ↑ Mannitol from 0.7 to 2.1 g/kg/24 h during HFO-TGI; ↑ Norepinephrine infusion rate from 0.24 to 0.29 μg/kg/min & Thiopental bolus of 250 mg during CMV2  ↑ HT saline from 0.11 to 0.33 g/kg/24 h & ↑ Norepinephrine infusion rate from 0.29 to 0.31 μg/kg/min during CMV1; Crystalloid bolus of 300 mL during HFO-TGI | 1: Death of septic shock and MOF at 7 days post-enrolment |
| **7 / study day 1**  **7 / study day 2** | ICP>20 mmHg & CPP<60 mmHg during CMVBL; ICP>20 mmHg during first 8 hours of HFO-TGI; **RMs of HFO-TGI session cancelled**^c^  ICP>20 mmHg and CPP<60 mmHg during CMV1; ICP>20 mmHg during the first 8 hours of HFO-TGI, and CMV2; CPP<60 mmHg & MAP most frequently within 65-69 mmHg during last 8 hours of HFO-TGI and CMV2 - refractory septic shock; **RMs of HFO-TGI session cancelled**^c^ | ICP=30 mmHg & CPP=42 mmHg (during CMV1); ICP=29 mmHg (while CPP≥60 mmHg), during HFO-TGI  ICP=26 mmHg & CPP=39 mmHg (during CMV2), with MAP=65 mmHg; maximal ICP=27 mmHg (during CMV2), with CPP≥45 mmHg & MAP>70 mmHg | ↑ Thiopental infusion rate from 6.0 to 7.0 mg/kg/h &  ↑ Norepinephrine from 0.27 to 0.31 μg/kg/min during CMVBL; ↓ PaCO_2_ within 30-35 mmHg during HFO-TGI  ↑ Norepinephrine infusion rate from 0.31 to 0.35 μg/kg/min & addition of Vasopressin at 0.04 IU/min^d^ & ↑ Mannitol from 0.9 to 2.2 g/kg/24 h during CMV1; ↑ HT saline from 0.14 to 0.32 g/kg/24 h during CMV2; Crystalloid bolus of 500 mL during HFO-TGI and during CMV2 | 1: Death after an episode of thrombocytopenia-associated, extensive intra-ventricular hemorrhage at 16 days poat- enrolment |

| **Patient No. /Day of**  **Secondary insult** | **Type, protocol sub-period, and duration of insult** | **Worst ICP and/or CPP values recorded during the designated study day sub-period(s)** | **Treatment of insult** | **GOSE^a^** |
| --- | --- | --- | --- | --- |
| **7 / study day 3** | ICP>20 mmHg throughout day 3; CPP<60 mmHg for <15 min during HFO-TGI and for <60 min during CMV2 | ICP=25 mmHg & CPP=54 mmHg (during CMV2) | ↑ Thiopental infusion rate from 7.0 to 8.0 mg/kg/h & ↑ Norepinephrine infusion rate from 0.35 to 0.38 μg/kg/min during CMV2 |  |
| **8 / study day 1**  **8 / study day 2**  **8 / study day 3** | ICP>20 mmHg for <60 min during both HFO-TGI and CMV2  ICP>20 mmHg and CPP mmHg <60 for <60 min during CMV1; **RMs of HFO-TGI session cancelled**^c^  CPP<60 mmHg for <15 min during HFO-TGI; ICP>20 mmHg & CPP<60 mmHg for <60 min during CMV2 | ICP=26 mmHg^i^ (during CMV2)  ICP=29 mmHg & CPP=55 mmHg (during CMV1)  CPP=54 mmHg (during HFO-TGI); ICP=23 mmHg & CPP=51 mmHg (during CMV2) | Addition of HT saline at 0.13 g/kg/24 h & ↑ of CMV minute ventilation by 1 L/min to improve PaCO_2_ control during CMV2  ↑ Propofol infusion rate to from 3.5 to 4.5 mg/kg/h & ↑ Norepinephrine infusion rate from 0.18 to 0.20 μg/kg/min during CMV1  ↑ Norepinephrine infusion rate to from 0.20 to 0.24 μg/kg/min for 15 min during HFO-TGI and then ↓ to 0.20 μg/kg/min after a Crystallid bolus of 500 mL; ↑ Norepinephrine infusion rate to 0.23 μg/kg/m and ↑ HT saline to 0.31 g/kg/24 h during CMV2 | 1: Death of septic shock and MOF at 9 days post-enrolment |
| **10 / study day 1** | ICP>20 mmHg & CPP<60 mmHg during CMV1; CPP<60 mmHg for <15 min during HFO-TGI; **RMs of HFO-TGI session cancelled**^c^ | ICP=41 mmHg^k^ & CPP=50 mmHg (during CMV1); CPP=58 mmHg (during HFO-TGI) | ↑ Norepinephrine infusion from 0.18 to 0.23 μg/kg/min & Thiopental bolus of 250 mg during CMV1 | 6: Upper moderate disability |
| **13 / study day 1**  **13 / study day 3** | ICP>20 mmHg & CPP<60 mmHg during CMV1 for <60 min; CPP<60 mmHg for <60 min during CMV2; **RMs of HFO-TGI session cancelled**^f^  CPP<60 mmHg for 5 min during CMV1; **First RM of HFO-TGI session not cancelled due to error in CPP calculation^h^; Error confirmation resulted in cancellation of second and third RM^h^** | ICP=22 mmHg & CPP=55 mmHg (during CMV1); CPP=56 mmHg (during CMV2)  CPP=58 mmHg (during CMV1) | ↑ Norepinephrine infusion from 0.20 to 0.22 μg/kg/min &  ↑ Propofol infusion rate from 3.0 to 3.5 mg/kg/h during CMV1; ↑ Norepinephrine infusion rate 0.22 to 0.24 μg/kg/mi during CMV2  CPP restored to ≥60 mmHg without additional therapeutic intervention | 8: Upper good recovery |

GOSE, Glasgow Outcome Scale Extended; ICP, intracranial pressure; CPP, cerebral perfusion pressure; MAP, mean arterial pressure; CMV, conventional mechanical ventilation; HFO, high-frequency oscillation; TGI, tracheal gas insufflation; CMV1 and CMV2, correspond to pre HFO-TGI CMV and post HFO-TGI CMV, respectively (see also Figure 1 of the main manuscript and corresponding legend); MOF, multiple organ failure; HT, hypertonic. CPP was calculated as the difference between MAP and ICP in all instances.

^a^, Determined at approximately 3 months after hospital discharge in survivors; data originate from patient follow-up records of the University-affiliated Department of Neurosurgery of Evaggelismos hospital.

^b^, Refers to a total physiological insult duration of 5-15 min during a designated sub-period of the study.

^c^, Recruitment maneuvers (RMs) of HFO-TGI cancelled due to ICP>25mmHg and CPP<60 mmHg during CMV1 (see also Methods of the main manuscript and Supplement to Results).

^d^, Defined as need for vasopressin at 0.02 to 0.04 IU/min in addition to norepinephrine for the maintenance of mean arterial pressure at >70 mmHg; in patient No. 2, dobutamine and vasopressin were added the last 3 hours of CMV2 of study day 1, vasopressin infusion rate was increased to 0.04 IU/min within the 3^rd^ to 7^th^ hour of CMV2 of study day 2, and then progressively reduced and discontinued within the 8^th^ to 10^th^ hour of CMV2 of study day 2; in patient No. 7, vasopressin was progressively reduced and discontinued within the 7^th^ to 9^th^ hour of CMV2 of study day 2; in both patients, study day 2 fluid balance was positive by 3.0-3.5 L.

^e^, Refers to a total physiological insult duration of 15-60 min during a designated sub-period of the study.

^f^, RMs of HFO-TGI session cancelled due to CPP<60 mmHg during CMV1 (see also Methods of the main manuscript).

^g^, RMs of HFO-TGI session cancelled due to ICP>25mmHg during CMV1 (see also Methods of the main manuscript).

^h^, First RM of HFO-TGI session not cancelled because of error in CPP averaging over the 5-min period of the physiological measurements of CMV1 (see also Methods of the main manuscript); this error resulted in an initially calculated, average CPP value of 60.4 mmHg, instead of the actually correct value of 58.4 mmHg; following error confirmation, the second and third RM of the HFO-TGI session were cancelled according to study protocol.

^i^, ICP perturbation associated with a post HFO-TGI PaCO_2_ rise of >5 mmHg relative to the end of the HFO-TGI session.

^k^, Reduced to ≤30 mmHg within <5 min of thiopental administration.

**
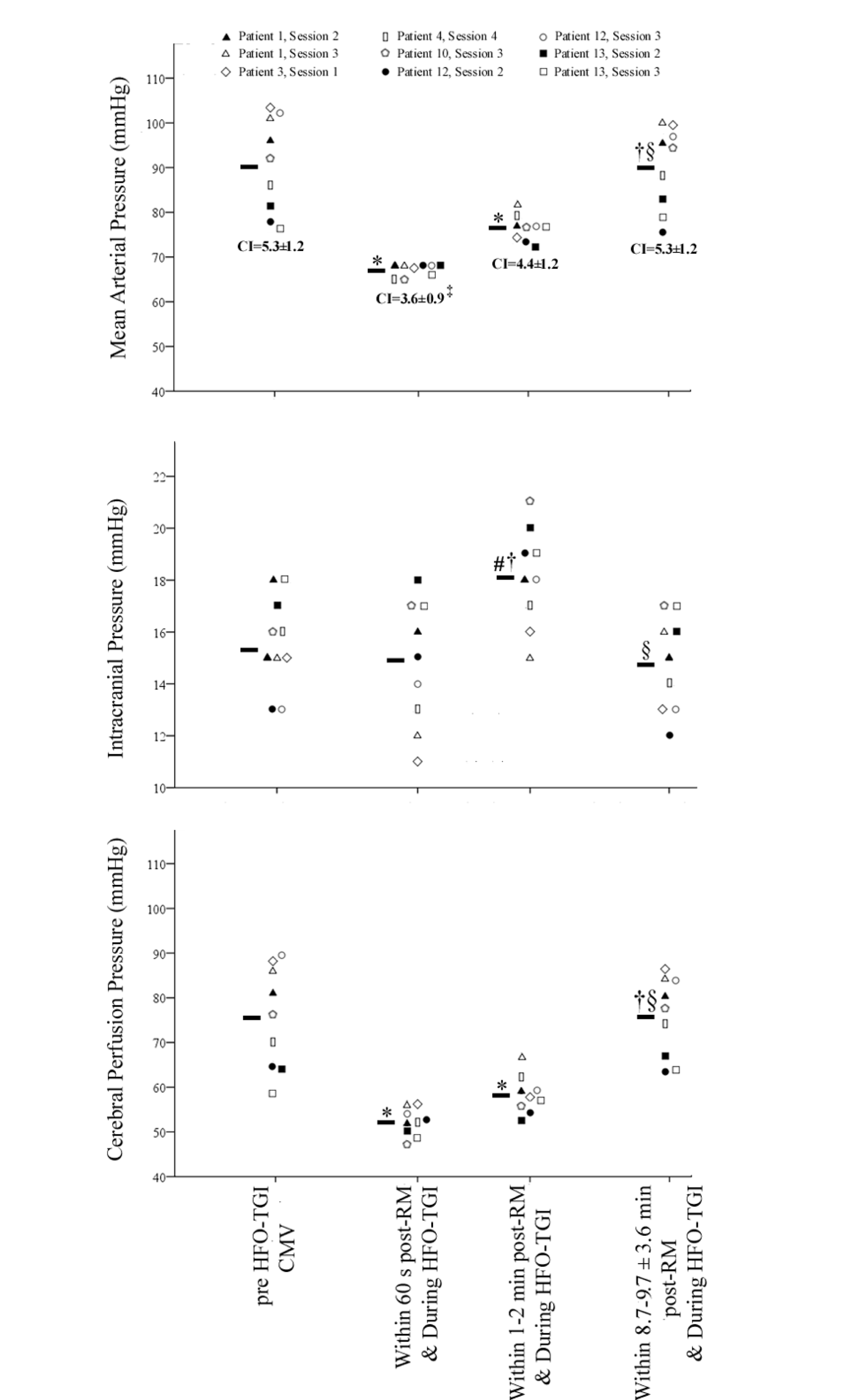
**

**Figure S1.** **Individual patient data corresponding to episodes of transient hypotension**.

CMV, conventional mechanical ventilation; HFO, high-frequency oscillation; TGI, tracheal gas insufflation; pre-HFO-TGI CMV, corresponds to either the baseline CMV period of study day 1, or the 60-min period that followed the 11 hour period of post HFO-TGI CMV of the preceding study day (see also Figure 1 of the main manuscript and corresponding legend); RM, recruitment maneuver; CI, cardiac index.

Top diagram: individual mean arterial pressure data and summary CI data (reported as mean±SD L/min/m^2^ body surface area), Middle diagram: individual intracranial pressure data, Bottom diagram: individual cerebral perfusion pressure data.

The episodes of hypotension (i.e. mean arterial pressure<70 mmHg) were observed within 2 min of the initiation of 9 sessions of HFO-TGI; recorded variable values were averaged over the period of the physiologic measurements of pre-HFO-TGI CMV (see also Figure 1 of the main manuscript), the first 60-s period after the first RM ("within 60 s post-RM"), the second 60-s period after the first RM ("within 1-2 min post-RM"), and the 60-s period that followed the time point of their restoration to within ±10% of the pre-HFO-TGI CMV values; the latter restoration of systemic and cerebral hemodynamics occurred at 8.7±3.6 min post-RM.

Horizontal bars represent mean of individual patient data. All reported data were compared with repeated measures analysis of variance for one within-subjects factor, followed by the Bonferroni correction for pairwise *post hoc* comparisons (see also Methods of the main manuscript).

*, P<0.01 vs. pre-HFO-TGI CMV

†, P<0.01 vs. "Within 60 s post-RM & During HFO-TGI"

§, P<0.01 vs. "Within 1-2 min post-RM & During HFO-TGI"

‡, P>0.05 and <0.10 vs. pre-HFO-TGI CMV and "Within 8.7-9.7±3.6 min post-RM & During HFO-TGI"

#, P<0.05 vs. pre-HFO-TGI CMV.
